# Investigation of quercetin and hyperoside as senolytics in adult human endothelial cells

**DOI:** 10.1371/journal.pone.0190374

**Published:** 2018-01-09

**Authors:** HyunTae V. Hwang, Darlene Thuy Tran, Michelle Nicole Rebuffatti, Chin-Shang Li, Anne A. Knowlton

**Affiliations:** 1 Molecular & Cellular Cardiology, Cardiovascular Division, Department of Internal Medicine, University of California-Davis, Davis, CA, United States of America; 2 Division of Biostatistics, Department of Public Health Sciences, University of California-Davis, Davis, CA, United States of America; 3 VA Medical Center, Sacramento, CA, United States of America; 4 Pharmacology Department, University of California-Davis, Davis, CA, United States of America; Niigata Daigaku, JAPAN

## Abstract

Quercetin has been reported to act as a senolytic by selectively removing senescent endothelial cells, and thus it would seem quercetin could revolutionize the field of gerontology. However, given quercetin's narrow therapeutic index reported in work done with human umbilical vein endothelial cells (HUVECs), we hypothesized that quercetin is not innocuous for non-senescent adult human vascular endothelial cells at concentrations that have been reported to be safe for proliferating HUVECs. Furthermore, we investigated quercetin 3-D-galactoside (Q3G; hyperoside), an inactive quercetin derivative that needs to be cleaved by beta-galactosidase overexpressed in senescent cells to release quercetin, as a potential safer senolytic. We compared the effectiveness of quercetin and Q3G in primary human coronary artery endothelial cells (HCAEC), which are adult microvascular cells. We found that quercetin caused cell death in non-senescent endothelial cells at a concentration that has been reported to selectively remove senescent cells, and that Q3G was not cytotoxic to either young or senescent cells. Thus, in primary adult human endothelial cells, quercetin and Q3G are not senolytics. Earlier work reporting positive results was done with HUVECs, and given their origin and the disparate findings from the current study, these may not be the best cells for evaluating potential senolytics in clinically relevant endothelial cells.

**New and noteworthy:**

Previously, quercetin has been reported to be a senolytic, a drug that selectively removes senescent cells, in HUVECs. However, we found neither quercetin nor Q3G was effective as a senolytic for adult human endothelial cells.

## Introduction

Quercetin is a flavonoid found in significant quantities in our diet with beneficial effects, including anti-thrombotic, anti-inflammatory, and anti-neoplastic properties [1–4]. It is an excellent antioxidant that scavenges many naturally occurring reactive oxygen species, including O_2_^-^ and ONOO^−^, and it facilitates zinc trafficking into cells, which in turn functions as an antioxidant [5, 6]. However, quercetin has been reported to induce cell type-specific cytotoxicity *in vitro*, where quercetin was relatively harmless to murine thymocytes and human lung embryonic fibroblasts at 100 μM, but significantly increased cell death was observed in human umbilical vein endothelial cells (HUVECs) at the same concentration [7, 8]. Despite this, following the finding that clinically relevant concentrations of glutathione completely suppressed quercetin's mutagenicity, and that no significant harm was observed in animals fed quercetin, it was determined to be safe for human consumption [9].

Quercetin 3-D-galactoside (Q3G), also known as hyperoside, is a natural derivative of quercetin produced by *Hypericumperforatum L*. (St. John's Wort) [10]. Q3G is structurally identical to quercetin, except for a galactoside group attached through an *O*-glycosidic bond that can be cleaved by beta-galactosidase to liberate quercetin [11]. Like quercetin, Q3G is bioactive with its anti-oxidant properties, even when it is not pre-processed by beta-galactosidase, and has other beneficial functions including inhibiting the growth of several parasites, lowering cholesterol, and fostering cardioprotection after ischemia [10, 12]. In addition, although quercetin-induced cytotoxicity has been reported in non-senescent HUVECs, Q3G is much safer for these cells, yet still confers protective anti-oxidative effect [13]. The major focus of quercetin research has been on its anti-oxidative effects. However, a groundbreaking new role for quercetin has been recently proposed, which may also extend to the quercetin derivative Q3G, based on recently reported findings that quercetin has senolytic properties, or the ability to selectively remove senescent cells. This would be an important development given the contribution of senescent cells to many of the deleterious changes of aging, including increased inflammation [14].

The *in vivo* development of cellular senescence, where cells halt normal function, irreversibly cease dividing, and secrete damaging inflammatory factors, has been proposed to be one of the major drivers of aging [15]. Cellular senescence is characterized by several prominent biochemical and functional changes, including flattened and enlarged cell morphology, increased lysosomal beta-galactosidase activity, and inflammatory factor secretion [15, 16]. The idea of cellular senescence contributing to the aging process is supported by the finding that senescent cells accumulate in aging organisms and at sites of age-related dysfunction, such as atrophic skin, osteoarthritic lesions, and atherosclerotic plaques [17].

Recent work reporting quercetin's potential as a senolytic used irradiation-induced senescent HUVECs, but HUVECs, which are derived from the umbilical vein of newborns, are far removed from aging adult human arterial vascular endothelial cells (EC). Not surprisingly, important differences have been found between adult EC and HUVEC [18–21]. Furthermore, quercetin’s low therapeutic/toxic ratio in the HUVEC study [14] raised the possibility that quercetin could significantly injure non-senescent cells. It was unclear whether the proliferation of non-senescent cells could be compensating for some of the quercetin-mediated cell death, thus masking its toxicity to the young cells at the lower concentrations found to be selectively cytotoxic to senescent cells. In the current study, we used adult human coronary artery endothelial cells (HCAEC), which are microvascular cells, as a relevant model, and generated two groups of cells from them to better understand the effect of quercetin: EP (early passage; young) and SEN (senescent), as a model of an aging tissue.

Given the known differences between adult EC and HUVECs, we hypothesized that quercetin would exhibit nonspecific cytotoxicity to adult EC. We investigated the effect of quercetin on EP vs. SEN HCAEC, and whether the SEN group was more susceptible to quercetin toxicity, as had been seen in irradiation-induced senescent HUVECs [14]. Furthermore, we tested whether Q3G, an inactive pro-drug that generates quercetin when cleaved by beta-galactosidase overexpressed in senescent cells, would more selectively remove senescent cells, and thus be a safer senolytic.

## Materials and methods

### Cell culture

HCAEC from three different adult human female donors, frozen at passage 3, were purchased [Cell Applications (San Diego, CA, USA) Lot#2228, Cell Applications Lot#2827, Lonza (Mapleton, IL, USA) Lot# 396592]. Donor information for the cells, supplied by the vendors, is as follows: #2228 (21 years old, Caucasian female), #2827 (17 years old, Hispanic female), #396592 (32 years old, Caucasian female). The cause of death and medical history for the donors is personal protected information, and thus unavailable. Common causes of death in younger females are accidents (30–40% of deaths), suicide and homicide (9–18%) [22]. Endothelial cell identity has been confirmed by uptake of acetylated LDL and presence of Factor III. Mycoplasma testing was negative. The cells were cultured in VascuLIFE^®^ VEGF-MV (Lifeline Cell Technology; Frederick, MD, USA), containing 5% FBS, 5 ng/mL FGF, 50 μg/mL ascorbic acid, 10 mM L-Glutamine, 15 ng/mL IGF-1, 5 ng/mL EGF, 5 ng/mL VEGF, and 0.75 U/mL heparin sulfate. Antibiotics and hydrocortisone were not used. Cells were seeded at 3000 cells/cm^2^ for each passage. Culture medium was changed every two days, and the cells were kept in a 5% CO_2_ humidified incubator at 37°C.

### Establishment of EP and SEN cells

The HCAEC were thawed and allowed to proliferate (Passage 1). After 4 days, they were passaged (Passage 2), and were then cryogenically stored with 2X freezing buffer [40% FBS (GE Life Sciences; Marlborough, MA, USA), 40% VascuLIFE^®^ VEGF-MV (Lifeline Cell Technology), 20% DMSO (Sigma-Aldrich; St. Louis, MO, USA), 100 IU Penicillin-Streptomycin (Thermo Fisher Scientific; Waltham, MA, USA)]. At the time of an experiment, a vial was thawed and cultured for 4 days to allow time for recovery. The subsequent passage (Passage 3) was considered EP, where treatment of quercetin or Q3G began 2 days after this point for 48 hours.

To establish SEN cells, the cells were serially passaged every 4 days and re-plated at the density of 3000 cells/cm^2^, thereby gradually decreasing the cells' proliferation from the initial rate of 10- to 30-fold increase between passages to less than 2-fold increase. When the proliferation rate had decreased to the point where the cell number failed to double within 4 days, the subsequent passage was considered SEN. Once the passage at which the cells undergo senescence was identified, a different batch of cells was harvested 3- to 4- passages before reaching this point and was cryogenically stored until desired for experiments. We opted to freeze the cells at 3- to 4- passages before reaching senescence, rather than 1 passage prior like the EP cells, because we were concerned the senescent cells may be more prone to injury by the freezing process at this late passage due to their characteristic enlarged cell size. Cellular senescence was confirmed through visual inspection for the characteristic flattened and enlarged morphology, senescence-associated beta-galactosidase (SABG) staining, and decreased expression of Lamin B1 [23]. Thus, cellular senescence and SEN group were operationally, visually, and biochemically defined and established in our study.

### Quercetin and Q3G preparation and treatment

Quercetin (CAS# 117-39-5; Cat# 10005169) and Q3G (CAS# 482-36-0; Cat# 18648) were purchased from Cayman Chemical (Ann Arbor, MI, USA). The stock solutions for these chemicals were prepared in DMSO at the concentration of 20 mM, aliquoted into small airtight tubes, and were stored in either liquid nitrogen or -80°C. Quercetin has been reported to degrade once it is in solution [24]. To prevent this, each of the stock aliquots kept in -80°C was used within a month of preparation and discarded after the single thaw for the experiment, as storing at low, freezing temperatures was recommended by other investigators and one supplier (Abcam) [25–27].

**Fig 1:**
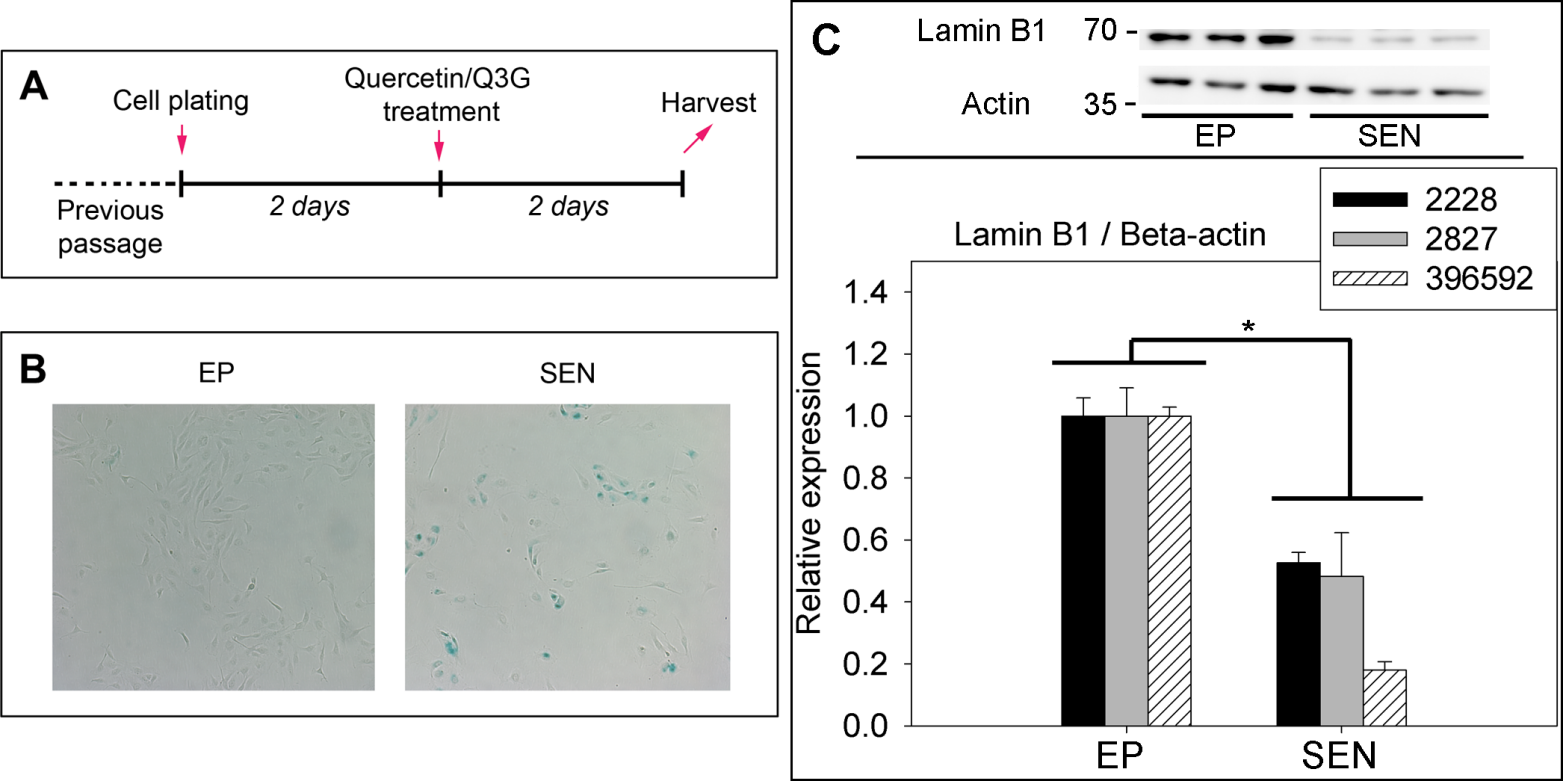
A) The experimental timeline. Cells were treated with either quercetin or Q3G for 48 hours before assays. B) A representative SABG staining comparing EP and SEN cells. Many of the SEN cells show characteristic flattened morphology, and are stained blue due to increased SABG activity. C) Representative image of the western blot and densitometry data for Lamin B1. *p<0.05 vs. baseline (N = 3 samples/group, per donor), T-test.

For treatment of HCAEC, quercetin and Q3G were first diluted in the culture medium to create working stock solutions, and then these solutions were mixed in different proportions with the culture medium to achieve the concentrations needed for the experiments. The blank culture medium had the same concentration of DMSO as the working stock solutions, so all cells were treated with the same concentration of DMSO. These working solutions were prepared immediately prior to treatment, and were applied to subconfluent cells two days after cell seeding. Based on the preliminary data on cell viability with quercetin/Q3G treatment (data not shown), both EP and SEN cells were treated for 48 hours at the end of their last passages (Fig 1A) at 0.21 mL per cm^2^ of growth area.

### Western blot

Whole-cell lysates were prepared by scraping the cultured cells in radio-immunoprecipitation assay (RIPA) buffer containing protease and phosphatase inhibitors (Sigma Aldrich; St. Louis, MO, USA; Cat# P8340, Cat# P0044). The samples were sonicated to facilitate cell lysis, and then centrifuged at 500g for 5 minutes to remove unlysed cells. The samples were separated on a 10% SDS-PAGE gel under reducing and denaturing conditions, and then transferred to nitrocellulose membranes for western blot, as previously described[28]. The following antibodies were used for probing: Lamin B1 (Cell Signaling; Danvers, MA, USA; Cat# 15068), and beta-actin (Sigma Aldrich; St. Louis, MO, USA; Cat# A2228)

### Senescence-associated beta-galactosidase staining

SABG staining was carried out according to a previously published protocol [29]. Briefly, EP and SEN cells were fixed with a formaldehyde-glutaraldehyde buffer, and incubated with the X-gal staining solution overnight at 37°C. The cells were then washed with DPBS (Dulbecco's phosphate-buffered saline) and kept in the buffer during light microscopy imaging to prevent desiccation and deformation of cells.

As an alternate assay to confirm the findings for this study, the SABG stained cells were manually counted. To minimize bias, counting of the cells was done by researchers blinded to the cell treatment group. For counts, a well in the culture plate was positioned on the microscope stage without looking into the eyepiece, preventing subconsciously choosing a particular field of view to assay. Then, without re-positioning the plate, both stained and non-stained cells visible in the field of view were counted.

### Cell proliferation assay

The DNA content-based fluorescence assay, CyQUANT^®^ Cell Proliferation Assay Kit (Cat# C7026), was purchased from Thermo Fisher Scientific. HCAEC were grown and treated with quercetin/Q3G in black 96-well tissue culture microplates with a clear bottom. Then, t0 (immediately before treatment) and Day 2 (48 hour treatment) plates were washed with DPBS (containing Ca^++^ and Mg^++^, to prevent cell detaching) and stored at -80°C. Both plates were treated with the supplied DNA-sensitive fluorescence dye and analyzed at the same time with a microplate reader (Molecular Devices; Sunnyvale, CA, USA), following the manufacturer's protocol.

### Live-Dead assay

Cell viability was measured with the LIVE/DEAD^®^ Fixable Green Dead Cell Stain Kit for 488 nm Excitation (Cat# L23101, Thermo Fisher Scientific). EC were grown on six-well tissue culture plates, and the cells were washed twice with DPBS containing Ca^++^ and Mg^++^ immediately prior to quercetin/Q3G treatment to remove the small amount of floating dead cells and debris that routinely occur with cell culture. After 48 hours of treatment, the following from each well were collected and centrifuged at 500 rcf for 5 minutes to obtain mixtures of live and dead cells: conditioned culture medium, cells detached with trypsin-EDTA, and DPBS-well washes. Then, the assay was performed according to the manufacturer's protocol for flow cytometry, with formaldehyde fixation. Non-fragmented whole cells were identified and gated with the forward scatter (FSC) parameter (S1A and S1B Fig).

### Statistics

**Fig 2:**
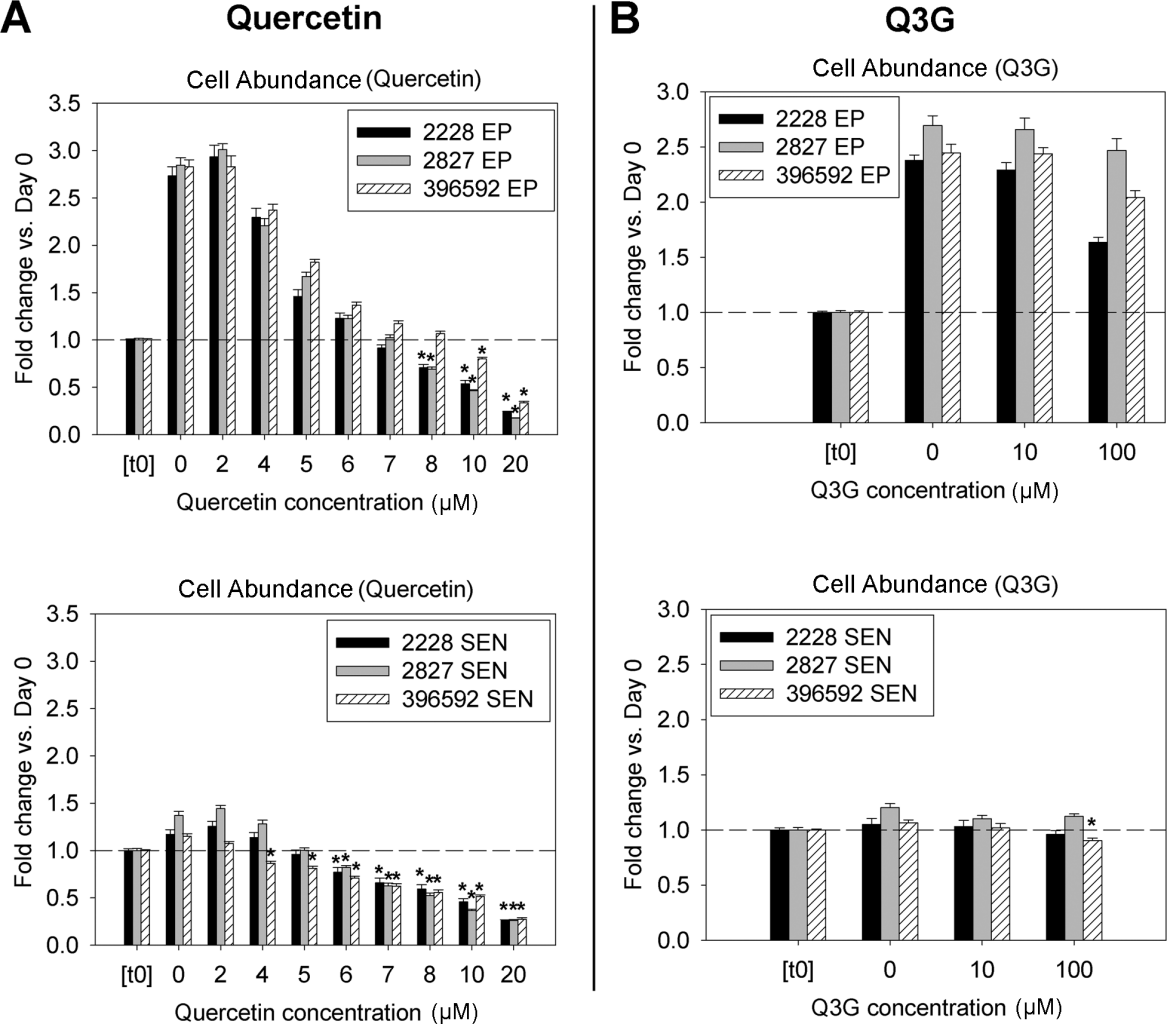
Relative cell abundance following quercetin A) and Q3G B) treatment of EP and SEN from three different donors. Cell proliferation was measured 48 hours after treatment, and was compared against the baseline count of cells frozen just prior to beginning treatment (t0). Values significantly lower than the t0 are indicated by asterisks. *p<0.05 vs. baseline (N = 16 wells/group, per donor), Kruskal-Wallis and Mann-Whitney U tests.

**Fig 3:**
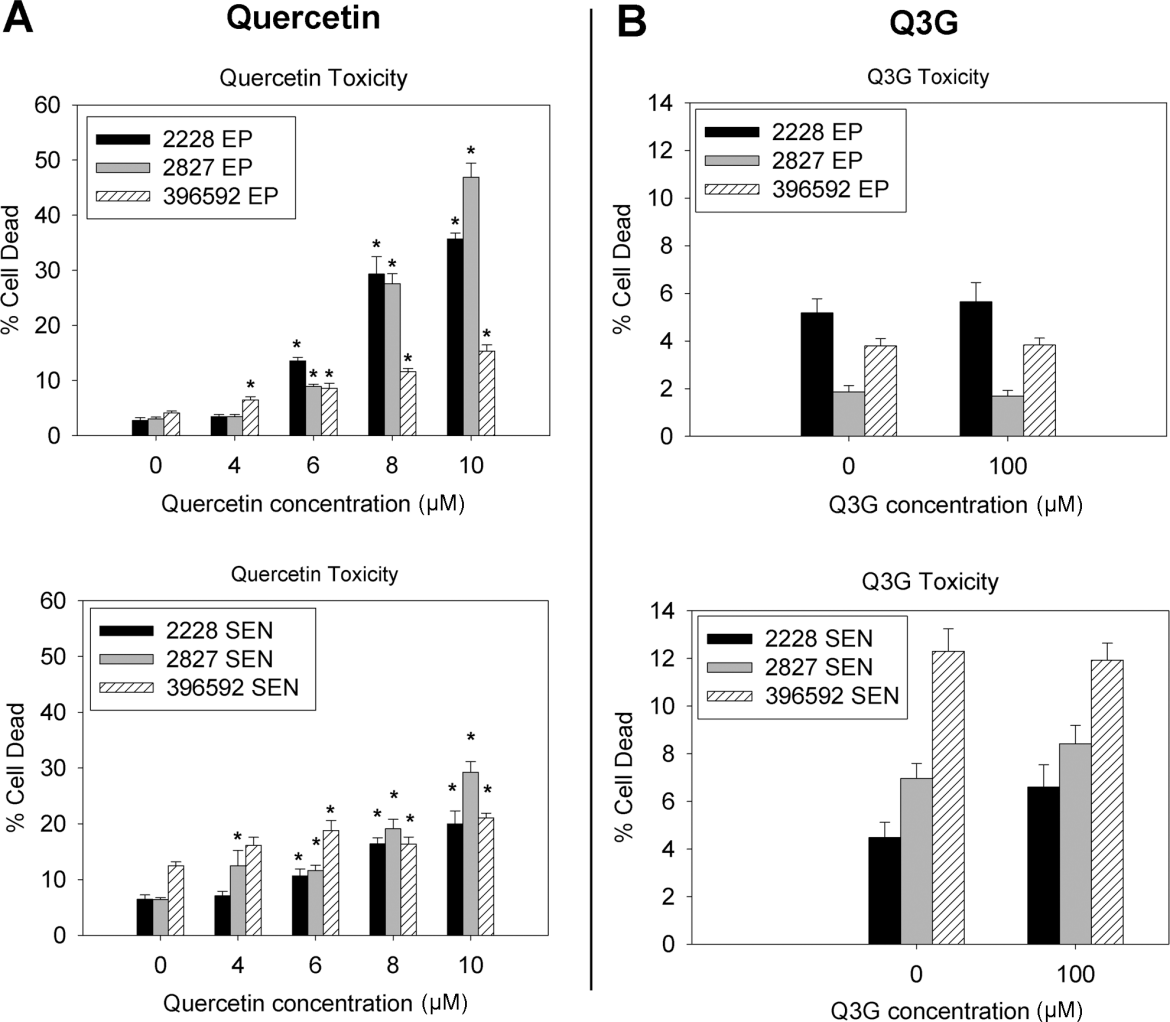
Live-Dead assay for quercetin (A) and Q3G (B) treatment of EP and SEN cells from the same three different donors. Data reflect the percentage of dead cells in response to different concentrations of quercetin or Q3G. N ≥ 6 wells/group, per donor. Approximately 1000 non-fragmented cells were scored for each N. *p<0.05 vs. baseline value. One-way ANOVA was used for Q3G data, which are normally distributed. Kruskal-Wallis and Mann-Whitney U tests were used for quercetin treatment to account for non-normality.

**Fig 4:**
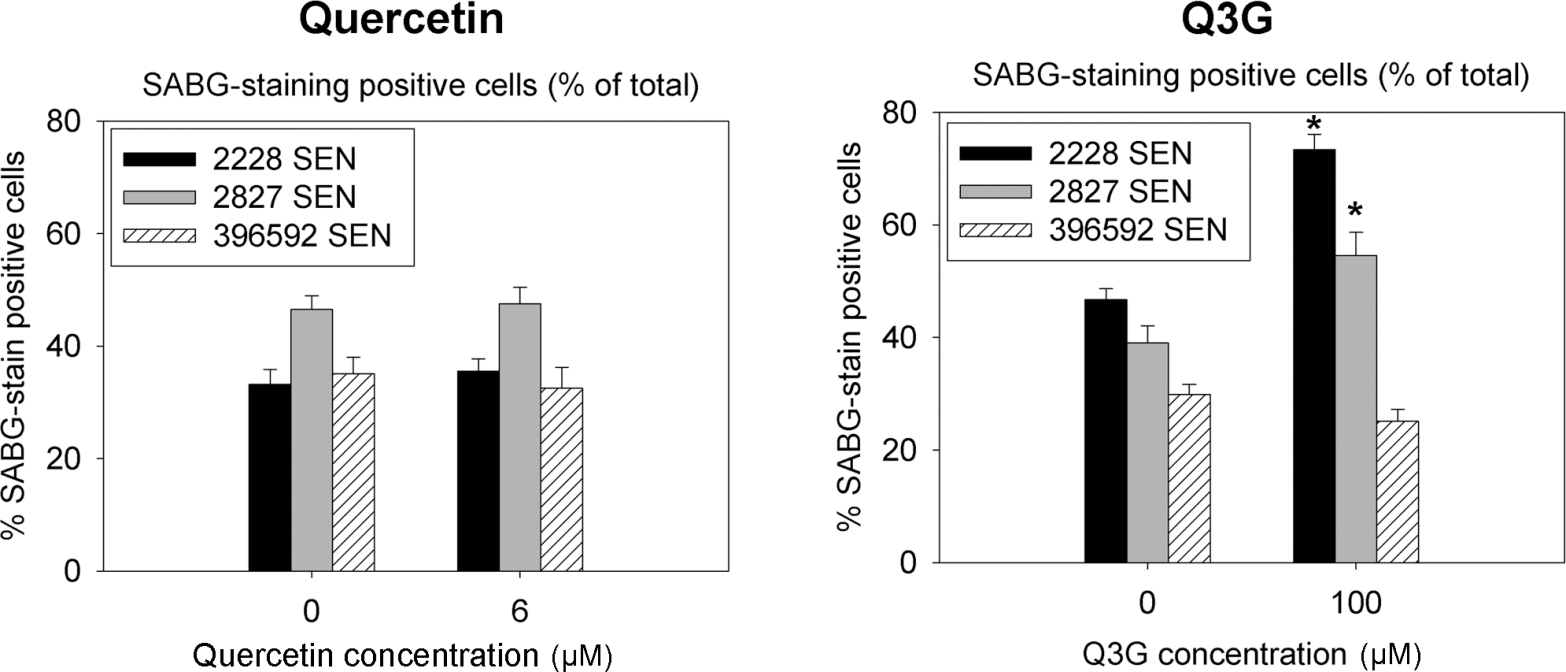
Manual quantification of SABG stain-positive cells. SEN cells from the same three donors were treated with 6 μM quercetin and 100 μM Q3G followed by SABG staining. Manual counting was done to assess percent of SABG positive cells. *p<0.05 vs. baseline value (N = 12 wells/group, per donor, where each N ranged from 40 to 160 cells). T-test was used to determine statistical significance between control and test groups for each donor.

Multiple groups were compared using a one-way ANOVA, and Kruskal-Wallis and Mann-Whitney U tests were used for the whole quercetin or Q3G data if data in any group were not normally distributed (Figs 2 and 3). Normally distributed data consisting of only two groups per donor was compared by T-test (Figs 1C and 4). SigmaPlot (Systat Software; San Jose, CA, USA) and SPSS v.23 (IBM; North Castle, NY, USA) were used for all statistical analyses. Data is reported as the mean ± the standard error of the mean (SEM). A p < 0.05 was considered significant.

## Results

For all experiments, HCAEC isolated from three different female donors were used to account for individual genetic variations, and only the data points that were positive for all three donors were considered relevant. For example, if only two of the three donors showed toxicity at a certain concentration of quercetin or Q3G, then this concentration was not considered damaging.

### Establishment of EP and SEN cells

Similar numbers of passages (10 to 13) were required to reach senescence with characteristic morphologic changes and expression of SABG (Fig 1B), as well as decreased expression of Lamin B1 (Fig 1C), for the three donors. For each donor, the passage number for onset of senescence was highly reproducible. The experimental time line is summarized in Fig 1A. A small subset of cells was still proliferating at this stage, as indicated by the cell proliferation assay, discussed below (Fig 2), but the overwhelming majority of the cells showed the characteristic morphological changes associated with cellular senescence, including the enlargement and flattening of the cells.

### Anti-proliferative effect of quercetin, but not Q3G, in EP and SEN cells

The proliferation of ECs was assessed following treatment with either quercetin or Q3G (Fig 2). The cell counts for both EP and SEN cells were increased in the respective 0 μM (vehicle only) groups compared to baseline values at Day 0 ("t0"), reflecting their natural proliferation over 48 hours (Fig 2A). Cell counts decreased with the increasing concentration of quercetin, consistent with a dose-dependent anti-proliferative effect, which has been previously reported in various cell types [2]. However, when the cell counts dropped below the initial baseline value, this suggested that cell death had occurred. At 6 μM quercetin, SEN cell numbers for all three donors were significantly lower than their respective baseline values (p ≤ 0.004). EP cells did not manifest quercetin toxicity until 10 μM, consistent with SEN cells' increased sensitivity to quercetin's cytotoxicity. In contrast, Q3G did not reduce the cell proliferation even at 100 μM, except for SEN cells from one donor, where a small, but significant dip in cell counts occurred (Fig 2B).

### Quercetin toxicity to EP and SEN EC

The Live-Dead assay was performed with flow cytometry to directly assess cell death with quercetin/Q3G treatment (S1A and S1B Fig; Fig 3). This assay revealed quercetin-mediated toxicity in EP cells at a lower concentration than our previously determined threshold of 10 μM (Fig 2A): EP cells from all three donors had a significant increase in cell death when treated with 6 or more μM of quercetin (Fig 3A). Thus, the EP cells' apparent resistance to quercetin-induced cell death exhibited in the cell proliferation assay (Fig 2) was likely due the lost cells being replaced by proliferation, thereby masking quercetin's toxicity. As for SEN cells, results similar to EP cells were observed (Fig 3), where all three donors also had significantly elevated cell death beginning at 6 μM, suggesting non-specific toxicity of quercetin. Q3G did not affect cell death at any concentration in EP or SEN cells (Fig 3B). Thus, the Live-Dead assay clearly demonstrates an increase in cell death in EP cells, as well as SEN cells, rather than selective culling of SEN cells.

### Quercetin and Q3G treatment on the prevalence of senescent cells

To directly assess whether senescent cells treated with quercetin or Q3G decreased in number, we used direct counting of SABG stain-positive cells by researchers blinded to treatment groups (Fig 4). SEN cells were treated with 6 μM quercetin based on the cell proliferation data, which had indicated that this concentration was nontoxic for EP cells, but reduced the number of SEN cells (Fig 2). For Q3G, 100 μM was used to determine if it was capable of reducing the number of senescent cells. The SEN cells treated with 6 μM quercetin showed no reduction in SABG stain-positive, senescent cells (Fig 4). Paradoxically, we found an increase in the population of senescent cells for two of the three donors treated with 100 μM Q3G, but as not all donors showed the same response, we deemed this inconclusive. Thus, neither quercetin nor Q3G reduced the prevalence of senescent cells.

## Discussion

Previously, quercetin was reported to be a senolytic in irradiation-induced senescent HUVECs [14]. HUVECs are derived from the umbilical cord of newborn babies, and for a long time were the only model of primary human EC; however, these cells are not the best model of diseases associated with human arterial aging. HUVECs have been shown to differ substantially from primary endothelial cells derived from adult human vasculature [18–21]. In the current study, we investigated whether quercetin is a senolytic in adult EC, and evaluated whether Q3G would be a more selective senolytic. Our key findings are that quercetin at a concentration that reduced SEN EC also caused significant EP EC cell death (Fig 3), and that there was no evidence of senescent cell-specific cell death mediated by quercetin (Fig 4). Thus, quercetin is not a selective senolytic in adult human arterial endothelial cells, where both EP and SEN cells responded similarly to quercetin's toxicity. In contrast, Q3G had no significant cytotoxicity at all (Figs 2B, 3B and 4).

In this study, we performed three different assays to evaluate quercetin and Q3G. Our SEN cells are a mixture of senescent cells positive for SABG staining (25 to 45%) and near-senescent cells, which are beginning to show the characteristic morphological changes related to cellular senescence, but are SABG stain-negative and slowly proliferate (Figs 1, 2 and 4). As cellular senescence induced by replication, which is a physiologically relevant stress, and ionizing radiation, have similar, but nevertheless distinct phenotypes [30], we think this is a more representative model of aging tissue.

SEN cells were more sensitive to quercetin treatment than EP ECs. EP cells did not begin to show cytotoxicity until 10 μM quercetin, which differed substantially from the previous work with HUVECs, where proliferating cells tolerated up to 20 μM quercetin [14]. HCAECs' increased sensitivity to quercetin's cytotoxicity compared to HUVECs is consistent with previous studies that reported HUVECs' disparate responses compared to adult EC [18, 20].

To determine if quercetin's toxicity in EP cells was masked by their proliferation, we directly examined the number of dead cells. Quercetin has been reported to be capable of inducing both necrosis and apoptosis. [31] The Live-Dead assay, which captures both types of cell death, showed that EP cell death was increased (Fig 3) at a concentration that appeared to be safe based on the cell proliferation data (Fig 2). Importantly, both EP and SEN cells exhibited signs of increased cell death at the same quercetin concentration. Thus, quercetin toxicity was not selective.

In the Live-Dead assay, we evaluated only non-fragmented whole cells by flow cytometry and thus may have undercounted cell death. Therefore, we manually counted senescent cells treated with quercetin or Q3G (Fig 4) to determine if a selective decrease of senescent cells occurred in the SEN cell mixed population. This manual count showed that neither quercetin nor Q3G selectively decreased senescent cells.

### Quercetin and the heat shock response

An important detrimental property of quercetin is inhibition of the heat shock response (HSR) [32]. The HSR is an important cytoprotective cellular response conferred by the induction of heat shock proteins (HSPs). The primary role of HSR is to mitigate proteotoxic damage stemming from a wide range of stresses, including heat, radiation, inflammation, ischemia/reperfusion injury, stretch, and reactive oxygen species [33–36]. However, the HSR is blunted with aging, as lower activity of HSF1, the primary transcription factor for the HSR and HSP expression, and HSF1-mediated HSR has been observed in senescent cells [37, 38], in the aging heart [28] and in aging humans [39, 40]. Given quercetin's direct effect on HSF1, which downregulates both its level and activation [32], treating older individuals, who would already have an impaired HSR, with quercetin either through high dose dietary supplements or intravenous administration as an anti-senescence treatment may have a significant downside, including accumulation of misfolded proteins [41].

### Senolytics and aging

The first study demonstrating the physiological benefits of selective removal of senescent cells utilized transgenic progeroid mice with a novel, inducible transgene, which would eliminate p16^Ink4a^-positive, senescent cells [42]. This selective removal of senescent cells was able to prevent key age-related dysfunctions in the mice, including lordokyphosis, muscle atrophy, and cataract development [42].

The first clinically relevant senolytics for humans were discussed in a study published in 2015: quercetin and dasatinib [14]. Since then, several additional senolytic agents have been reported [43–45], and the development of this class of drugs is ongoing. Recently, geldanamycin and tanespimycin, the inhibitors of heat shock protein 90 (HSP90), were identified as senolytics [45]. Inhibiting HSP90 leads to the activation of HSF1 and upregulation of HSPs [46–48], which is the opposite effect of quercetin. However, it is unclear if activation of HSF1 is necessary for the senolytic activity of geldanamycin and tanespimycin.

### Q3G as a selective alternative to quercetin

To circumvent quercetin's toxicity on healthy, non-senescent cells, we investigated Q3G, a derivative of quercetin with limited toxicity to endothelial cells, which is processed by SABG enriched in senescent cells to release quercetin *in situ* [10–13]. Q3G could act as a selective prodrug in senescent cells. However, Q3G had no significant toxicity to either EP or SEN EC. The ability of Q3G to cross the cell membrane despite its large polar structure has been demonstrated by its *in vitro* bioactivity in treated cells [49], and Q3G has been found to be able to release quercetin through the activity of exogenous beta-galactosidase [11]. The lack of Q3G's toxicity in the current study may be due to Q3G being unable to enter the beta-galactosidase-rich lysosomes [16], or alternatively, Q3G being able to translocate to the lysosomes to release quercetin, which is further processed into an inert compound.

In conclusion, our results demonstrate that neither quercetin nor Q3G is effective as a senolytic for adult EC *in vitro*. The difference in the findings between our work and the results of Zhu et al. [14] may reflect the important differences between HUVECs and mature adult EC, as well as different models of senescence–radiation vs. replicative senescence, which is a more physiological model of aging. Given the disparity in the sources and phenotypes of the cells, HCAECs may be a more relevant model for studying senolytics than HUVECs.

## Supporting information

S1 Fig Representative plots for flow cytometry. –A) Representative scatter plots for flow cytometry with different concentrations of quercetin treatment. B) Representative scatter plots for flow cytometry with different concentrations of Q3G treatment. For both panels, the number at the lower left in the plot indicates the percentage of events within the gate compared to the entire events shown in the plot.
(TIF)

S1 File Raw data. (XLS)
