# Supplementary figures and images for: Investigation of quercetin and hyperoside as senolytics in adult human endothelial cells
Source: PLoS One. 2018 Jan 9;13(1):e0190374. doi: 10.1371/journal.pone.0190374 (PMC5760026; doi:10.1371/journal.pone.0190374)

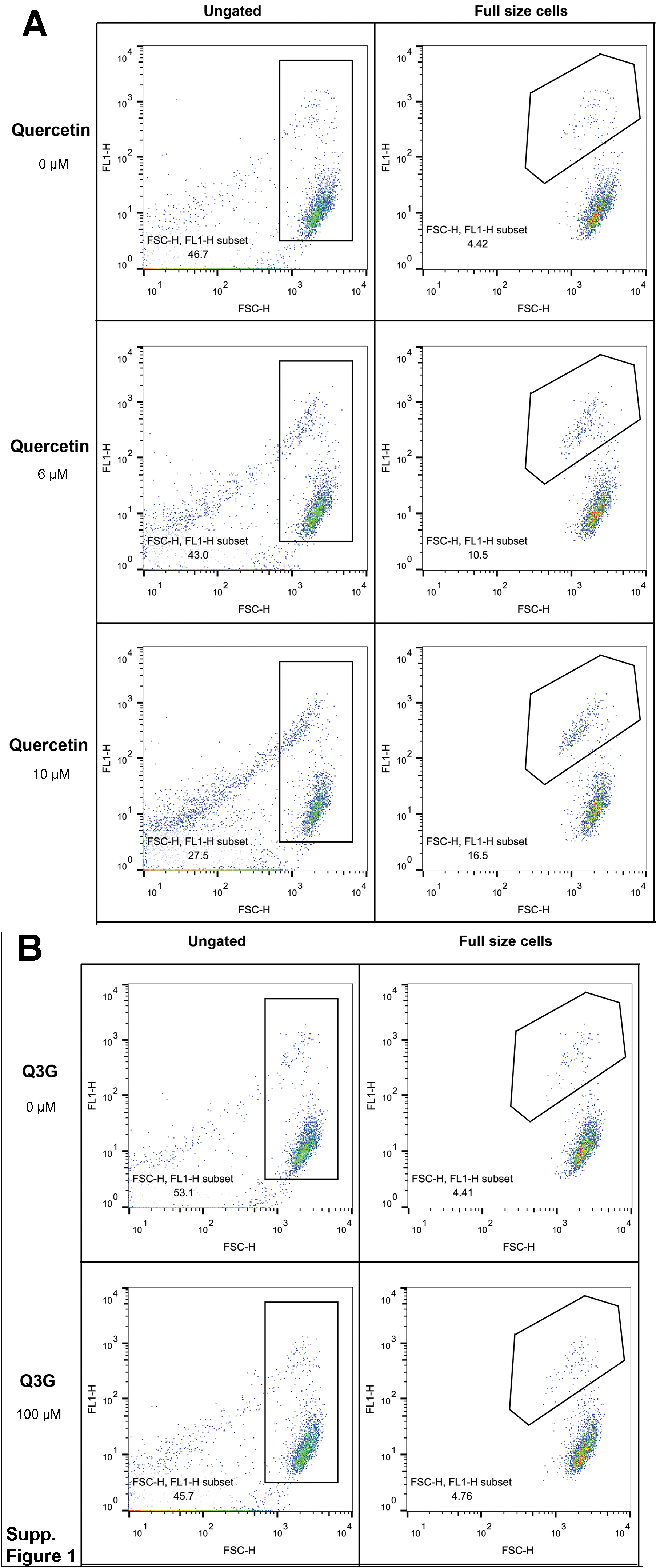

Supplement: S1 Fig — –A) Representative scatter plots for flow cytometry with different concentrations of quercetin treatment. B) Representative scatter plots for flow cytometry with different concentrations of Q3G treatment. For both panels, the number at the lower left in the plot indicates the percentage of events within the gate compared to the entire events shown in the plot. (TIF) [file pone.0190374.s001.tif]
